# Supplementary figures and images for: Inducible degradation of the Drosophila Mediator subunit Med19 reveals its role in regulating developmental but not constitutively-expressed genes
Source: PLoS One. 2022 Nov 29;17(11):e0275613. doi: 10.1371/journal.pone.0275613 (PMC9707739; doi:10.1371/journal.pone.0275613)

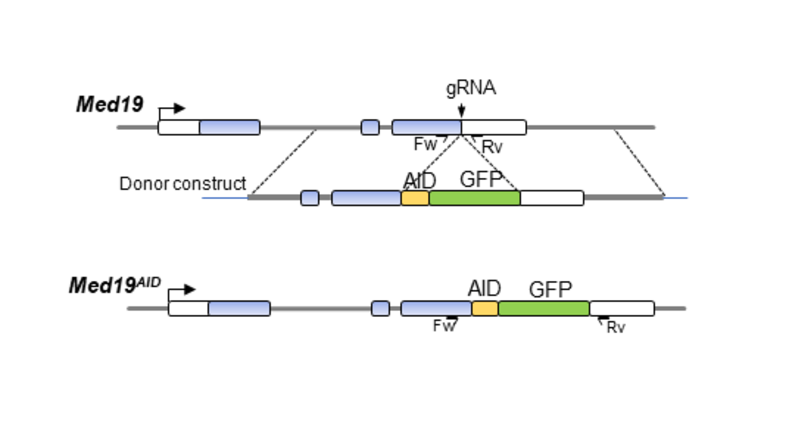

Supplement: S1 Fig — The donner construct (middle) highlights the nature of the homology arms used to introduce the AID-GFP cassette by homologous recombination. Position of the PCR primers used to characterize the wt vs 19AID allele is shown. (TIF) [file pone.0275613.s001.tif]

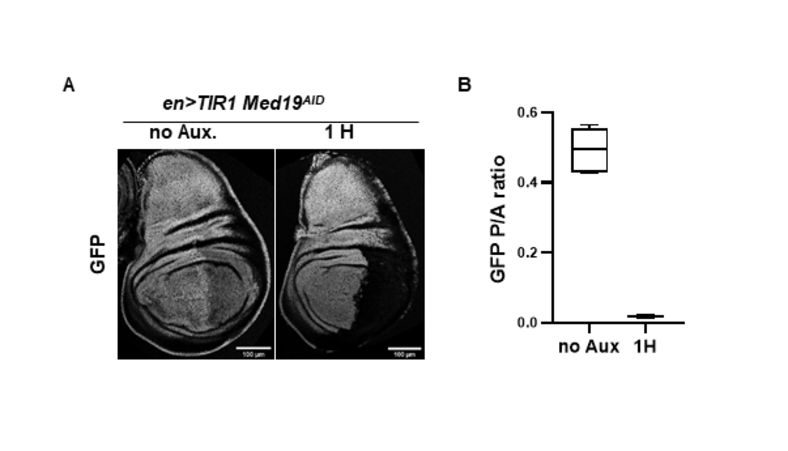

Supplement: S2 Fig — (A) Confocal images of wing discs dissected from en>TIR1 Med19AID larvae either fed for one hour with NAA (1H), or mock treated (no auxin). (B) Box plot showing the Med19AID depletion level calculated as the posterior to anterior GFP intensity ratio in the wing pouch of en>TIR1; Med19AID wing imaginal discs (n = 5) obtained and imaged as in (A). (TIF) [file pone.0275613.s002.tif]

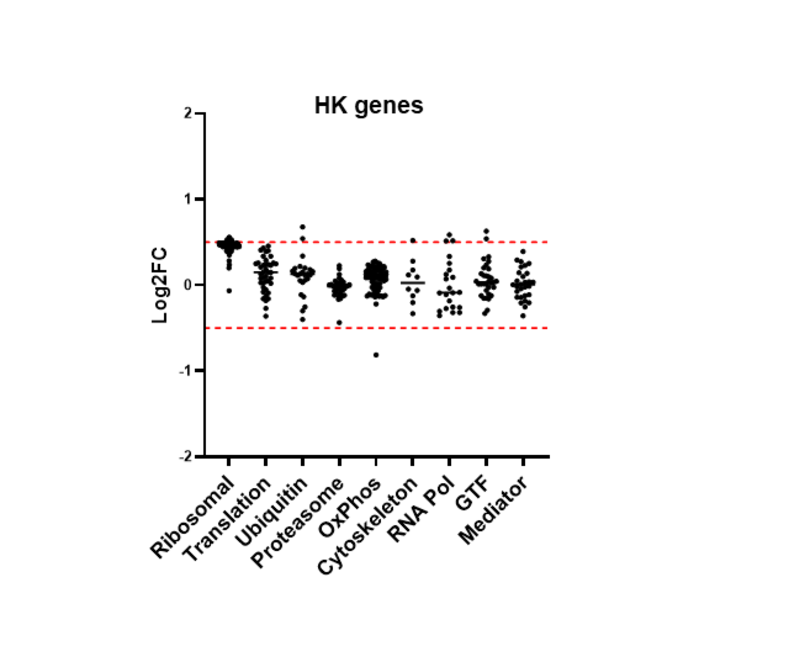

Supplement: S3 Fig — Scatter plot analysis of the expression fold change (Log2FC) of genes belonging to several families of housekeeping functions (x axis, list detailed in S3 Table). The red dashed lines mark Log2FC = +/ - 0.5. (TIF) [file pone.0275613.s003.tif]

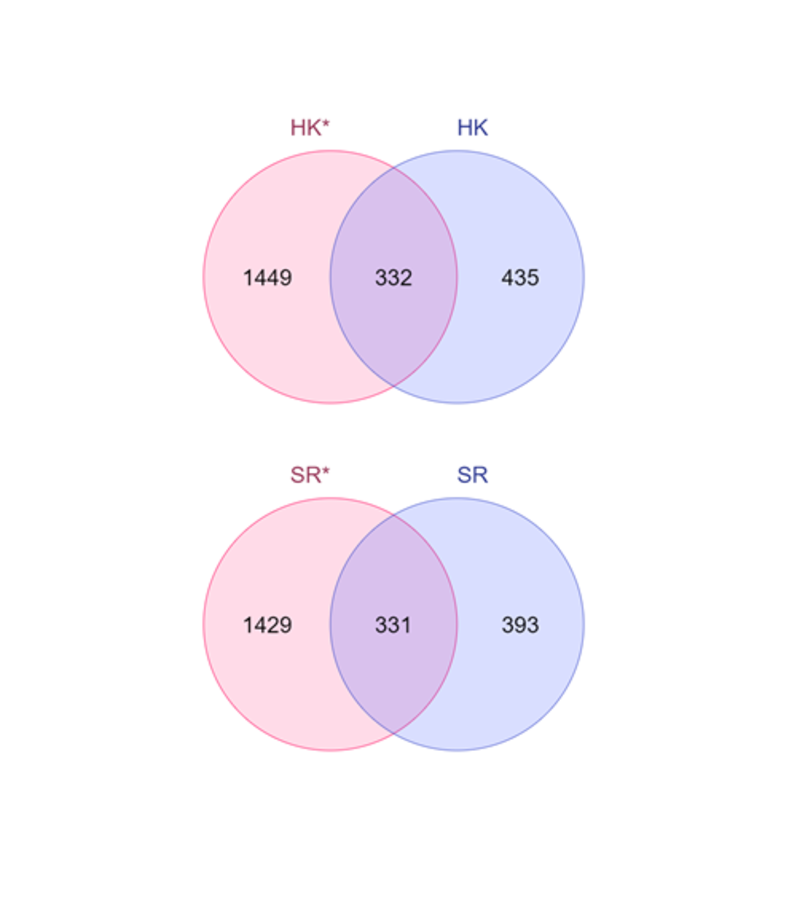

Supplement: S4 Fig — (TIF) [file pone.0275613.s004.tif]

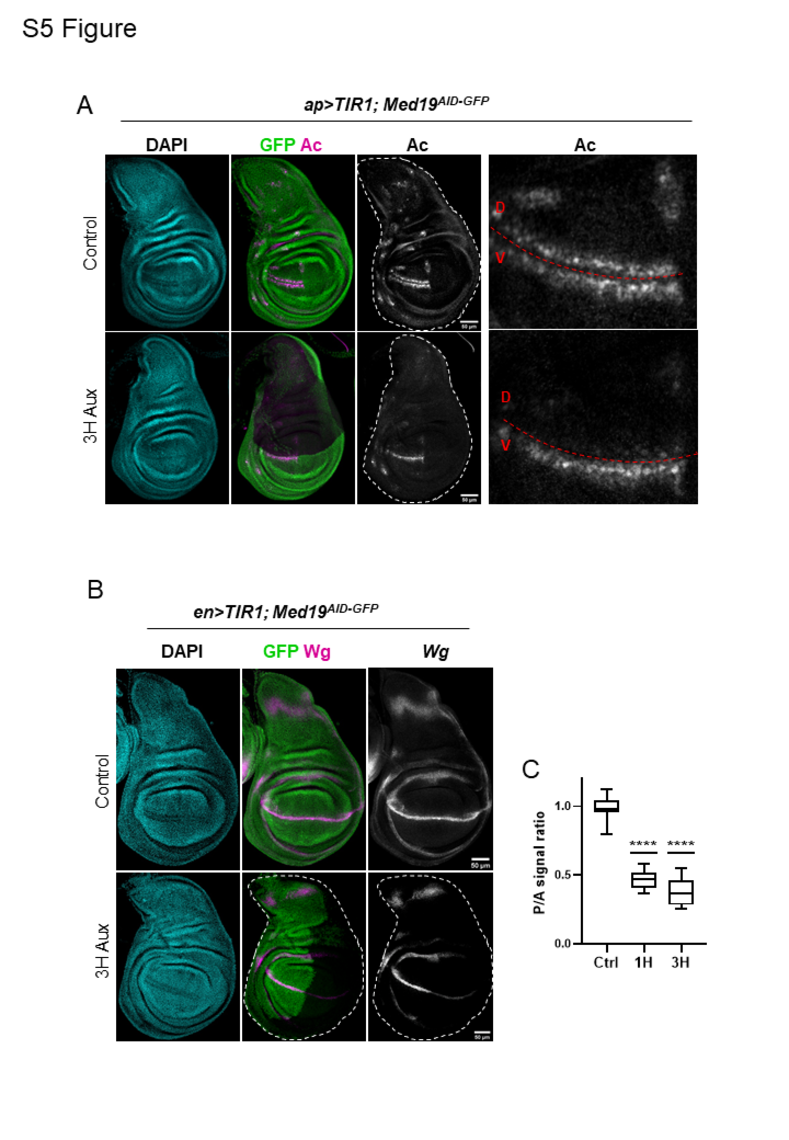

Supplement: S5 Fig — (A) Confocal images of ap>TIR1; Med19AID wing imaginal disc immunostained for Ac and GFP (Med19AID detection). Upper row: Control wing disc in which Med19AID degradation did not occur (no driver). Bottom row: Wing disc in which Med19 degradation was induced for 3 hours in the dorsal compartment, using an apterous driver. Right column: Magnification in the anterior wing pouch Ac staining pattern emphasizing the loss of Ac expression in the dorsal row of cells of the anterior wing margin when Med19AID degradation is triggered in the dorsal compartment of the wing disc. (B) en>TIR1; Med19AID wing imaginal discs immunostained for Wg and GFP (Med19AID), in which the degradation of Med19AID was operated with the en> driver for 3 hours (bottom row), or not triggered (control, upper row). (TIF) [file pone.0275613.s005.tif]

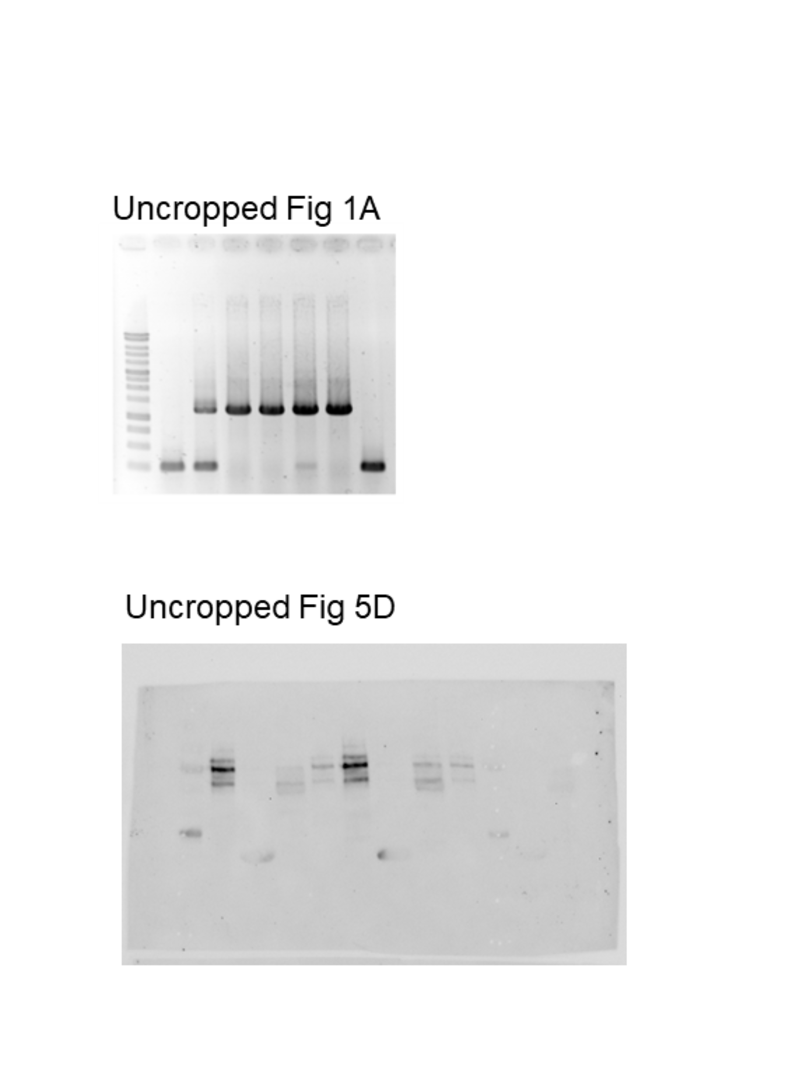

Supplement: S6 Fig — (TIF) [file pone.0275613.s006.tif]

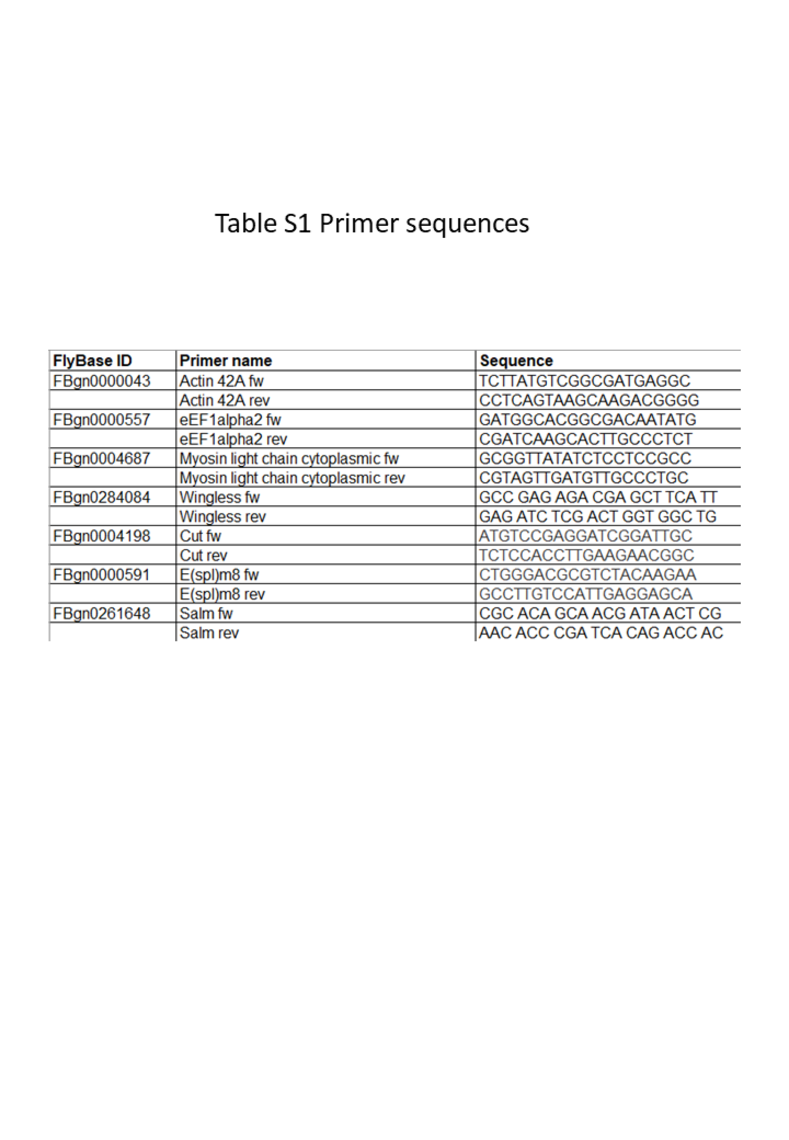

Supplement: S1 Table — (TIF) [file pone.0275613.s007.tif]

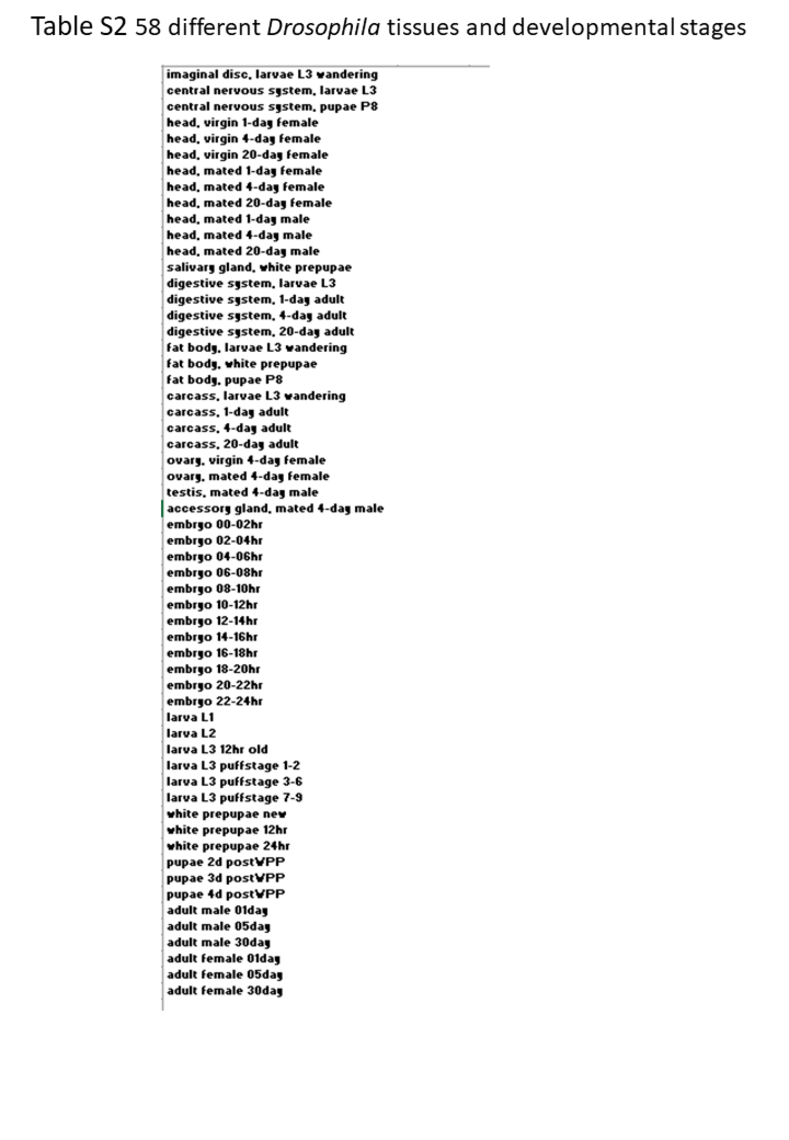

Supplement: S2 Table — (TIF) [file pone.0275613.s008.tif]

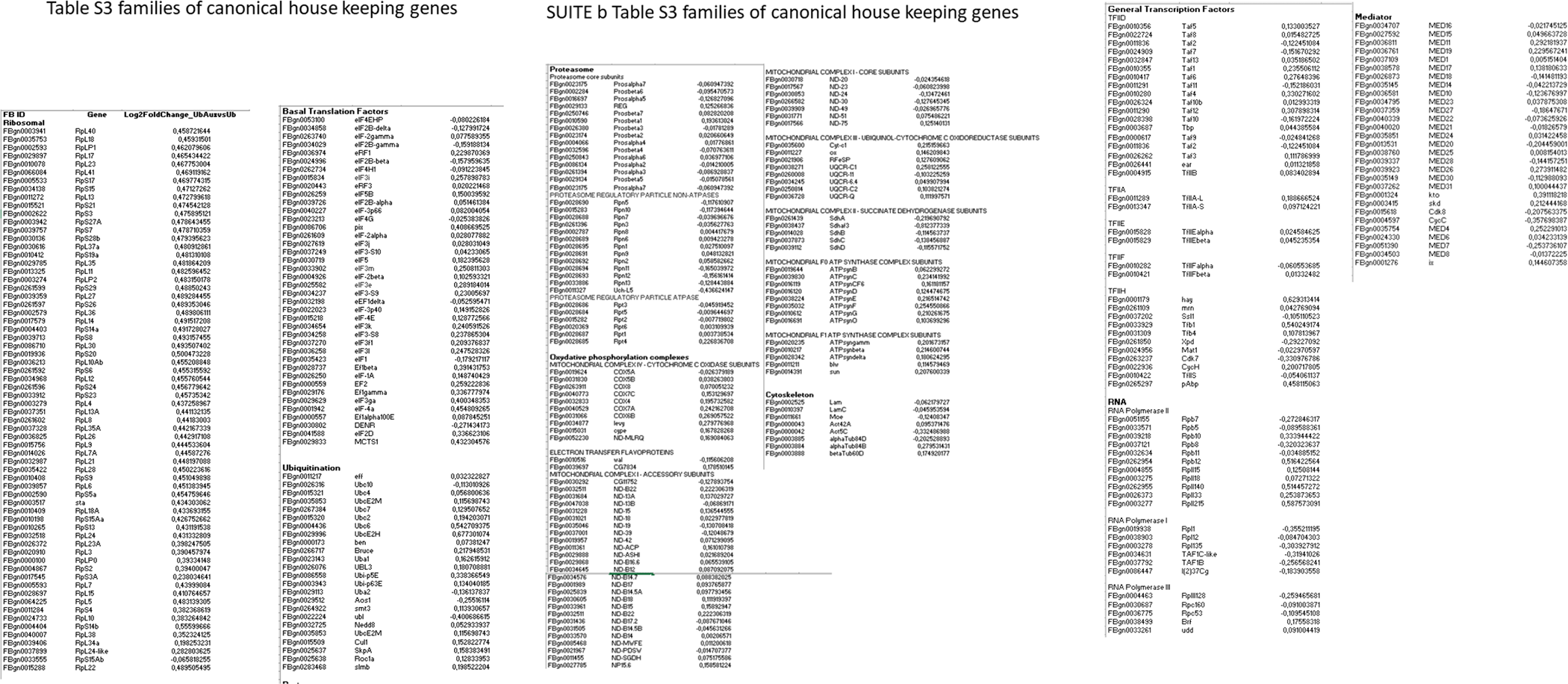

Supplement: S3 Table — (TIF) [file pone.0275613.s009.tif]

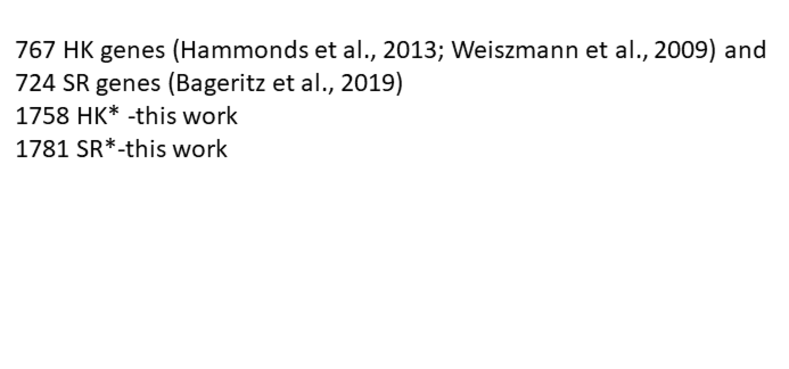

Supplement: S4 Table — (TIF) [file pone.0275613.s010.tif]

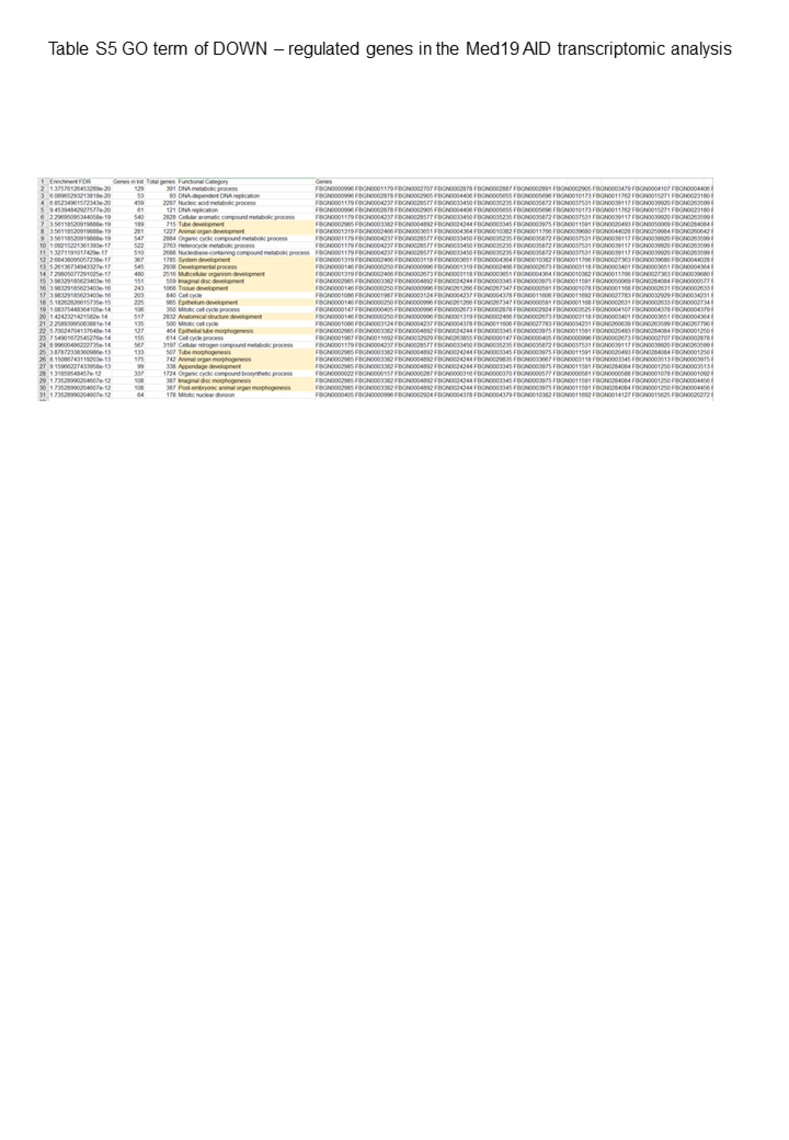

Supplement: S5 Table — (TIF) [file pone.0275613.s011.tif]
